# Learning from mistakes: analyzing incidents in a neonatal care unit^*^

**DOI:** 10.1590/1518-8345.2795.3121

**Published:** 2019-01-31

**Authors:** Louíse Viecili Hoffmeister, Gisela Maria Schebella Souto de Moura, Ana Paula Morais de Carvalho Macedo

**Affiliations:** 1 Hospital Moinhos de Vento, Porto Alegre, RS, Brazil.; 2 Universidade Federal do Rio Grande do Sul, Escola de Enfermagem, Porto Alegre, RS, Brazil.; 3 Universidade do Minho, Escola Superior de Enfermagem, Braga, Portugal.

**Keywords:** Patient Safety, Medical Errors, Neonatology, Nursing Care, Medication Errors, Quality of Health Care, Segurança do Paciente, Erros Médicos, Neonatologia, Cuidados de Enfermagem, Erros de Medicação, Qualidade da Assistência à Saúde, Seguridad del Paciente, Errores Médicos, Neonatología, Atención de Enfermería, Errores de Medicación, Calidad de la Atención de Salud

## Abstract

**Objective:**

to analyze incidents reported in a neonatal care unit.

**Method:**

a quantitative, cross-sectional and retrospective study with a sample of 34 newborns. Data were collected through a structured form, composed of two parts: sociodemographic/clinical characteristics of the newborns, and characteristics of the reported incidents. Data were collected from the institution’s computer system, in a period corresponding to 13 months, and analyzed by means of descriptive statistics.

**Results:**

the majority of the newborns were preterm (70.6%), male (52.9%) and born through caesarean section (76.5%). During the study period, 54 incidents were reported, totaling a frequency of 1.6 incident per newborn. It was found that 61.1% of incidents were related to medicines, 14.8% to accidental loss of tracheal tube and 9.3% to catheter obstruction.

**Conclusion:**

analysis of the reported incidents has shown that most incidents refer to the drug process. Information about the incidents can increase the perception of health professionals regarding the impact of their actions.

## RESUMO

**Objetivo:**

analisar os incidentes notificados em uma unidade de cuidados neonatais.

**Método:**

estudo quantitativo, transversal e retrospectivo, com amostra de 34 recém-nascidos. A coleta dos dados ocorreu mediante preenchimento de formulário estruturado, composto por duas partes: características sociodemográficas/clínicas dos recém-nascidos, e características dos incidentes notificados. Os dados foram coletados do sistema informático da instituição, em um período correspondente a 13 meses, sendo analisados por meio de estatística descritiva.

**Resultados:**

quanto às características sociodemográficas e clínicas, a maioria dos recém-nascidos era de prematuros (70,6%), do sexo masculino (52,9%) e que nasceu por meio de cesariana (76,5%). No período estudado foram notificados 54 incidentes, totalizando uma frequência de 1,6 incidente por recém-nascido. Encontrou-se que 61,1% dos incidentes estavam relacionados a medicamentos, 14,8% associados à perda acidental de tubo traqueal e 9,3% ligados à obstrução de cateteres.

**Conclusão:**

a análise dos incidentes notificados demonstrou que a maioria dos incidentes se refere ao processo de medicamentos. As informações sobre os incidentes podem ampliar a percepção dos profissionais de saúde em relação ao impacto das suas ações.

## resumen

**Objetivo:**

analizar los incidentes notificados en una unidad de cuidados neonatales.

**Método:**

estudio cuantitativo, transversal y retrospectivo, con muestra de 34 recién nacidos. La recolección de los datos fue completando el formulario estructurado, compuesto por dos partes: características sociodemográficas/clínicas de los recién nacidos y características de los incidentes notificados. Los datos fueron recogidos del sistema informático de la institución, en un período correspondiente a 13 meses, siendo analizados por medio de estadística descriptiva.

**Resultados:**

en las características sociodemográficas y clínicas, la mayoría de los recién nacidos era prematuros (70,6%), del sexo masculino (52,9%) y nacieron por medio de cesárea (76,5%). En el período estudiado fueron notificados 54 incidentes, totalizando una frecuencia de 1,6 incidentes por recién nacido. Se encontró que 61,1% de los incidentes estaban relacionados a medicamentos, 14,8% asociados a pérdida accidental de tubo traqueal y 9,3% ligados a obstrucción de catéteres.

**Conclusión:**

el análisis de los incidentes notificados demostraron que la mayoría de los incidentes se refiere al proceso de medicamentos. Las informaciones sobre los incidentes pueden ampliar la percepción de los profesionales de salud en relación al impacto de sus acciones.

## Introduction

Health care institutions have been concerned with inadequately performed procedures due to the high number of incidents during the care process. After more than 15 years of publication of the “To Err Is Human: Building a Safer Health System” report by the Institute of Medicine, recent studies have pointed out that the frequency of adverse events in health facilities has not reduced, although many actions aimed at patient safety and reduction of these events have been conducted^1^ ^-^ ^2^. The lack of safety in the care of patients can prolong hospitalization, increase hospitalization costs, generate additional treatments, extra tests and procedures, and irreparable damage to the health of individuals^3^. For these reasons, among others, providing quality and safety in healthcare has become a daily and arduous challenge for institutions.

The knowledge produced about the study of incidents is not only an important tool in the review of the care process, but also as a support in the planning of improvement actions. Thus, voluntary reporting of incidents and review of medical records are crucial procedures for managers and health professionals in the composition of a diagnosis of the quality of care provided and the areas that deserve greater attention.

In a recent study published in Portugal, the authors pointed out that knowing the characteristics of the population and the structure of the institutions where care is provided is essential for the development and implementation of strategies and solutions for the reduction of adverse events^4^. According to a study carried out in Argentina, the identification and analysis of adverse events are seen as key components of improvement programs in the area of patient safety. The authors further describe that each error, each adverse event, should be considered not only as a source of learning for health professionals, but also an opportunity to improve practices^5^.

The issue of safety, especially when associated with the occurrence of incidents in the hospital context, becomes a much more delicate subject when analyzed under the perspective of highly specialized care, as is the case of neonatal care. It is an area that presents great scientific and technological advances, achieving in the last years a greater understanding of the specificities of the newborns and, consequently, a reduction in infant morbidity and mortality. In a recent study, the authors report that “in neonatal intensive care units a single patient, sometimes an extreme premature, is manipulated by several professionals, which predisposes to an increased chance of suffering the consequences of an error”^6^.

With regard to neonatal care, some studies have been published with the purpose of measuring the occurrence of adverse events in this type of service. A study published in Argentina, with the objective of describing the epidemiology of adverse events in a neonatal population of Buenos Aires, found a relative frequency of clinical histories with the presence of at least one adverse event of 16.9%, being the occurrence of adverse event associated with hospitalization in the Intensive Care Unit, prolonged hospitalization, lower gestational age and lower birth weight^5^. 

In the United States of America, in a study conducted in a Neonatal Intensive Care Unit, the authors described a 74% of incident rate in hospitalized newborns, with the most frequent occurrences the infections associated with health care, accidental extubations, intravenous catheter infiltrations, skin rupture and intraventricular hemorrhage^7^.

Another study, conducted in Brazil, reported that 183 (84%) of the 218 newborns included in the investigation had suffered some type of adverse event. Of the 579 identified adverse events, with a rate of 2.6 adverse events per patient, 29% were thermoregulation disorders, 17.1% were glycemia disorders, 13.5% were hospital healthcare-related infections and, lastly, 10% referred to unscheduled extubation^8^. 

The incidence of errors and adverse events instigates health organizations worldwide to promote a culture based on the best and safer care, seeking to reach professionals of the various levels of care. It is essential to understand that safety practices need to be adapted to different populations, and economic, social and cultural contexts. There is a scarcity of investigations into the occurrence of incidents in the neonatal care unit, and a greater knowledge about this theme is necessary for the quality of care^6^.

In daily practice, it can be seen that the occurrence of incidents during the care process directly reflects the patient’s safety indicators, the quality of care and motivation of the professionals involved, although these facts are still devalued by many managers in health institutions. In view of the above, and the imminent importance of conducting studies that clarify this issue, the following question emerged: “What are the incidents that occur in a neonatal care unit of a private hospital in southern Brazil?”.

The objective of the present study was to analyze incidents reported in a neonatal care unit. This enabled knowing the incidents that occur in a neonatal care unit, taking into account the existence of few studies in Brazil on this subject. This knowledge can support health managers in justifying investments in institutional improvement actions and nurses in constructing safer work processes. 

## Method

This is a cross-sectional, retrospective, quantitative study conducted in a Neonatal Care Unit of a medium-sized private hospital located in the south of Brazil, with a total of 182 beds, 14 of them for the Neonatal Care Unit. Data collection took place in April and May 2017, through the completion of a structured form. The data were collected from voluntary notifications recorded in the institution’s computer system, retrospectively, in a period corresponding to 13 months, from May 2015 to May 2016.

The study population consisted of newborns admitted to the neonatal care unit of the referred hospital within the defined period for data collection, regardless of diagnosis and length of stay, who had undergone clinical or surgical treatment. Sampling was non-probabilistic, with individuals selected by convenience. This study were included the newborns who had a length of stay of more than 24 hours and who had at least one reported incident during their hospitalization. Newborns with of incomplete reports of incidents were excluded. Thus, the population was constituted by 340 newborns and the sample by 34 newborns. 

The form, developed by the researchers, was composed of two parts, namely: Part 1) data referring to the sociodemographic characteristics of the newborns, such as sex, age at birth (gestational age), age of hospitalization and age of discharge, type of delivery, birth weight and weight at discharge, Apgar score (in the first and fifth minutes of life), length of stay in the unit, date of birth, hospitalization date and discharge date, reason for hospitalization, origin and outcome of hospitalization; Part 2) data corresponding to incident reports, namely type of incident (care to which it relates), its classification, severity, avoidability, description of the incident and actions taken after the incident. In the first fortnight of data collection, a pilot test of the tool developed by the researchers was carried out and there was no need to change the instrument, so the data collected were used in the study.

The institution’s information technology team was asked to provide a list of newborns who had been hospitalized during the study period and who had at least one incident through recorded electronically. After providing this list, the data on the newborn and on incident reports were consulted in the institution’s computer system, allowing the form to be completed.

For the analysis of sociodemographic/clinical characteristics of newborns and frequency of incidents, descriptive statistics were used according to the variables collected. In order to analyze the quantitative variables with symmetrical distribution, we used mean and standard deviation, and for those with asymmetric distribution we used median and interquartile range. In categorical variables, absolute and relative frequencies were applied. The data were stored in the Excel program and analyzed by SPSS software, version 21.0.

The present study was approved by the Subcommittee on Ethics for Life and Health Sciences of the University of Minho under the number SECVS 020/2016, by the Evaluation Commission of Divina Providência Health Network, Brazil Platform, under the number CAAE 61164416.8.0000.5327 and by the Ethics Research Committee of the Graduate Group of the Hospital de Clínicas of Porto Alegre, under number 17-0010. The data obtained from the medical records were obtained through the signature of the Commitment Form on Data Use by the researcher in charge for the study. The study was exempt from the use of the Informed Consent Term Form due to the type of collection, and all data were collected from the information contained in the institution’s computer system.

## Results

The present study found that newborns who had suffered at least one incident during admission to the neonatal care unit represent 10% (n = 34) of the study population. Table 1 describes the results related to the sociodemographic and clinical characteristics of the newborns.

**Table 1:** Sociodemographic and clinical profile of newborns, Porto Alegre, RS, Brazil, 2017

**Variable**	**n=34**
**Gestational age (weeks) - mean ± standard deviation**	**34.6 ± 3.9**
Reason for hospitalization* - n (%)	
**Prematurity**	**22 (64.7)**
**Respiratory dysfunction**	**6 (17.6)**
**Severe asphyxia**	**2 (5.9)**
**Others**	**4 (11.8)**
**Sex - n (%)**	
**Male**	**18 (52.9)**
**Female**	**16 (47.1)**
**Type of birth - n (%)**	
**Ceserian section**	**26 (76.5)**
**Vaginal**	**8 (23.5)**
**Origin - n (%)**	
**Obstetric Center**	**29 (85.3)**
**Rooming-in**	**4 (11.8)**
**Home**	**1 (2.9)**

* Multiple answer

Regarding the age at birth, considering the gestational age according to the Capurro result, we found a mean of 34.6 weeks. When classifying newborns according to their age at birth, it was found that 1 (2.9%) of the newborns were post-term, 9 (26.5%) were at term, 10 (29.4%) were late preterm newborns, 8 (23.6%) were moderate preterm infants, 5 (14.7%) were severely premature infants and 1 (2.9%) were extreme preterm. Thus, most of the studied newborns were preterm infants, 24 (70.6%), a result that is in line with the reason for hospitalization.

Regarding the age of hospitalization, 30 (88.2%) newborns were admitted to the Neonatal Care Unit with less than 24 hours of life. With regard to the age at discharge, there was a median of 23 days (P25=14 - P75=56), which is in line to the length of stay in the unit. Regarding the length of stay, the median was 23 days (P25=14 - P75=56), and the newborn who remained the shortest period had been hospitalized for 10 days and the longest, for 102 days. The most frequent hospitalization period was 11 days, totaling 4 (11.8%) newborns.

The newborns were also evaluated regarding birth weight, weight at discharge from the Neonatal Care Unit and Apgar index in the 1st and 5th minutes of life. On birth weight, there was a median weight of 1910 grams (1645-2763), ranging from 715 grams to 4195 grams. Regarding the classification according to birth weight, 7 (20.6%) infants could be considered with extreme low birth weight and very low birth weight because they had a weight <1500g. On the other hand, newborns weighing between 1500 and 2499g, that is, low birth weight newborns, totaled 15 (44.1%). The remaining 12 (35.3%) newborns had a weight greater than or equal to 2500g. On the weight at discharge, a median of 2357 grams (2080-3308) was observed, ranging from 1945 grams to 5925 grams. As for Apgar scores, in the first minute, values between 0 and 10 were found, with a median of 8 (6-9); 15 (41.2%) of the newborns were evaluated with Apgar ≤ 7. At the fifth minute, the minimum value was 2 and the maximum 10, and the median was 8 (7-9); 9 (26.5%) newborns had Apgar ≤ 7. Another data collected was the outcome of newborn admission and, in this context, all the newborns studied had the outcome of hospital discharge.

Between May 2015 and May 2016, a total of 54 incidents were reported in the Neonatal Care Unit under study. These incidents occurred with 34 newborns, that is, some newborns suffered more than one incident during the period of hospitalization, totaling a frequency of 1.6 incident per newborn. 

Regarding the type of incident, 29 (53.7%) incidents were classified as incident without damage. The other incidents were classified as incident situations with damage, 14 (25.9%), and near miss situations, 11 (20.4%). Regarding the severity of the incidents, 14 (25.9%) incidents had moderate damage and 40 (74.1%) did not cause damage (incident without damage and near miss). All incidents occurred were considered preventable. 

The incidents were also analyzed on the type of associated care (Table 2).

**Table 2:** Characterization of reported incidents, Porto Alegre, RS, Brazil, 2017

**Variable**	**n=54**
**Care - n (%)**	
**Wrong drug administration**	**24 (44.4)**
**Omission of dose or infusion**	**6 (11)**
**Wrong or overdue reconstitution**	**5 (9.2)**
**Incorrect programming of the infusion pump**	**4 (7.4)**
**Overdose**	**3 (5.6)**
**Dilution in wrong quantity**	**2 (3.7)**
**Wrong solution installation**	**2 (3.7)**
**Wrong diluent**	**1 (1.9)**
**Wrong drug suspension**	**1 (1.9)**
**Accidental removal/loss of tracheal tube**	**8 (14.8)**
**Displacement of the tube or accidental extubation**	**7 (12.9)**
**Catheter traction**	**1 (1.9)**
**Catheter obstruction**	**5 (9.2)**
**Obstruction/resistance due to prolonged infusion**	**5 (9.2)**
**Wrong drug prescription**	**9 (16.7)**
**Overdose**	**4 (7.4)**
**Measurement unit error**	**2 (3.7)**
**Duplicate drug prescription**	**2 (3.7)**
**Wrong Patient**	**1 (1.9)**
**Wrong diet preparation/administration**	**2 (3.7)**
**Change of labels**	**2 (3.7)**
**Infiltrate venous access**	**1 (1.9)**
Infiltrate access infusing ATB*	**1 (1.9)**
**Skin injury**	**3 (5.6)**
**Skin breakdown**	**2 (3.7)**
**Tight ID bracelet**	**1 (1.9)**
**Hygiene/procedures**	**2 (3.7)**
**Prolonged tourniquet**	**1 (1.9)**
**Inadequate drug storage**	**1 (1.9)**

* Antibiotic

The data revealed that 24 (44.4%) of the incidents were related to wrong medication administration, 9 (16.7%) to wrong drug prescription, 8 (14.8%) to accidental removal/loss of tracheal tube, 5 (9.2%) to venous/arterial catheter obstruction and 3 (5.6%) to skin injuries. The other types of associated care totaled 5 (9.2%). 

On the actions taken after the occurrence of the 54 incidents, in 7 (12.9%) the immediate action was the preparation of a new medication or infusion, in 6 (11%) the incident was reported to a physician or nurse in the unit, in 5 (9.2%) there was catheter clearing and in 5 (9.2%) a new medical prescription was carried out. Other actions with smaller frequencies were performed, namely suspension of subsequent doses/infusions of the drugs; change in ventilation modality; change in the schedule of the next doses of medication; system alert on the incident and new medical prescription; passing new catheter or access; employee orientation; difficult reintubation; despised medicine; administering the missing dose of the drug; and dressing of a skin injury.

## Discussion

Research on the epidemiological profile of hospitalizations in neonatal units also found data related to the characteristics of newborns. In a Brazilian study, the authors described that 70% of the newborns had been born through cesarean delivery, 70% were premature (gestational age <37 weeks), 41.6% of the newborns had low birth weight (1500 to 2499 grams) and 26.6% very low birth weight (<1500 grams); also, 42% of these newborns received an Apgar score lower than seven in the first minute^9^. These same characteristics were described in another study, in which 53.14% of the newborns were male, 92.14% preterm (gestational age <37 weeks), 80.5% with low birth weight and 56% were born through cesarean delivery. In addition, prematurity was the main cause of hospitalization, with 77.04% of the admitted newborns^10^.

Some sociodemographic and clinical characteristics, such as length of stay, sex, type of delivery and Apgar score vary according to the studied population. On the other hand, characteristics such as low birth weight and prematurity, both regarding gestational age and the reason for admission, are related to the occurrence of adverse events and neonatal mortality, referenced in several studies^5^ ^-^ ^7^ ^,^ ^11^ ^-^ ^14^.

Regarding the frequency of incidents per newborn, some studies found in the literature describe this same data. A recent study of adverse events in pediatrics found that of the 3790 records examined, there were 414 adverse events (19.1 adverse events per 1000 patients/day) and 210 preventable adverse events (9.5 adverse events per 1000 patients/day), being more frequent in university hospitals and in chronic patients^15^. In a previously cited study, the authors analyzed 749 records using a record review procedure and found a total of 554 adverse events, which represents a rate of 0.74 events per records analyzed^7^. In another publication, researchers examined incidents reported voluntarily over a one-year period in eight neonatal care units and one pediatric unit of Dutch institutions and found 5225 incidents, of which 4846 were considered eligible for analysis, in 3859 hospitalizations, totaling 1.25 incidents due to hospitalization^16^. In another study, conducted in Brazil, 183 (84%) of the 218 newborns included in the investigation reported some type of adverse event. A total of 579 adverse events were identified, resulting in a rate of 3.16 adverse events per newborn^8^. This study presents higher results than those found in this investigation.

In relation to publications on neonatal incidents, there is a scarce number of studies on this subject, which highlights the need for further investigations with this approach and a deeper understanding of the characteristics of these incidents. There is a predominance of studies related to the occurrence of adverse events targeting the adult population, but a shortage of data focused on the pediatric population, especially newborns. In a recent study, the researchers reported that there is a gap in investigations on the occurrence of incidents, especially adverse events, in neonatal intensive care units^6^.

According to the results presented in a study conducted in Argentina, 65% of the adverse events found in the clinical histories of the newborns produced transient sequels without risk of death; however, half of the deaths that occurred were considered very likely to be preventable. Regarding the category, the incidents evidenced in 50% of the cases were related to the errors occurred during the monitoring of the clinical state or with the nursing care required by the neonates during hospitalization, for example the handling of catheters, accidental extubations, retinopathy of the preterm newborn, hemorrhages, transfusions, among others^5^.

In a study that investigated incidents involving mechanical ventilation and intravascular catheters in neonatology registered in a voluntary reporting system, the authors reported that of all reported incidents, 533 out of 1306 (41%) were linked to mechanical ventilation and intravascular catheters, particularly on incorrect configurations and connections, unplanned removal, mechanical failure, occlusion, and prolonged use. Severe, moderate and mild damage were reported, with 55% of incidents classified as human error^17^. In a current study that prospectively analyzed intubations in a neonatal care unit, the authors found during the investigation period 273 intubations with available data, of which 107 were intubations with adverse events. The increase in the number of intubation attempts and emergent intubations were predictors of adverse events^18^.

Most of the incidents analyzed in the present study were related to the medication, totaling 61.1% of the notifications. In a study recently published, the researchers found in a neonatal unit 511 reports of adverse drug-related events over a seven-year period, resulting in an incidence of 32.2 drug-related adverse events per 1000 days, with 39.5% of prescription errors, 68.1% of administration errors and 0.6% were adverse drug reaction^19^. 

When incidents are perceived, immediate actions are usually taken in an attempt to repair or minimize damages. In a study carried out in the United States, the authors reported in their research that patients, after suffering a medication error, required more constant monitoring or increased length of hospital stay (40.9%), onset/change in drug therapy (31.8%), increase in the number of tests (21.8%) or impairment of airways/resuscitation (1.3%). They also reported that of the 2706 reported reports, 48% reported that the error was first reported to the employees who made the mistake, 17.5% to employees who had been involved in the error, and only 8.7% cases the physician was informed^20^.

A reliable way of knowing the factors that cause the errors and that reduce the quality and safety of the care provided is through a detailed analysis of the incidents that occurred. A more in-depth knowledge of the incidence and characteristics of incidents, as well as the continuous monitoring of the occurrence of these errors, could help improve the quality of health care for the neonatal population^12^. In addition, actions are needed to prevent incidents, which include the continuous training of all professionals and the development of practices directed to the whole system, including the technical and organizational environment^17^. 

The present study chose to perform the search for incidents retrospectively and through electronic, anonymous and voluntary notifications in the computer system of the chosen institution. In a recent Brazilian publication, the researchers also chose to use information directly extracted from the databases of the studied institutions in order to avoid errors resulting from manual transcripts of information^21^. 

Although voluntary reporting of incidents is not considered the most effective way to detect adverse events, it is still the mechanism used by most health institutions. This is due to the fact that this tool is easily available to professionals, is a source of information that sometimes provides detailed descriptions of the facts, and assists in the review of processes. Underreporting of incidents is one of the reasons that prevents its effective voluntary use as a research tool on patient safety. 

Other strategies for detecting adverse events have been described in the literature, such as the active search for incidents in medical records, the use of triggers and the development of automated systems. In an American study, the authors identified 116 drug-related adverse events out of 10,104 drugs administered, through an automated adverse event detection system that, when compared to current practice (incident reports or trigger tools), showed a significant improvement of 4.3% to 85.3% (p = 0.009) at detection sensitivity. In addition, the new system demonstrated potential to reduce patient exposure to damage from 256 minutes to 35 minutes^22^.

The theme of patient safety and the study of incidents in neonatal care units is still a poorly explored area of knowledge with few studies described in the literature. The knowledge produced in the present study about the type, frequency, severity and causes of the reported incidents contributed with information that clarifies the magnitude of the incidents in the studied unit and that corroborate the other few published studies. In addition, knowing the reported incidents provided professionals and managers with support in choosing priority areas and actions for the development of improvements, since they could reflect on the mistakes most commonly made and valued by the teams. 

Voluntary notifications were the only source of incident identification, being a limitation of this study. Thus, it may have restricted the amount of information about them and reduced the scope of the investigation.

## Conclusion

It was evidenced that 10% of newborns admitted to the unit had undergone at least one incident during the investigation period, which points to the existence of flaws in care routines. The newborns studied were mostly premature babies, born from cesarean delivery, coming from the obstetric center, and having a birth weight of less than 2500 grams. Regarding the incidents, 54 reported errors were found in the IT system of the institution, 53.7% of which were classified as incidents without damages and 25.9% as an incident with moderate damage. 

The vast majority of the incidents were related to the therapeutic processes and had as immediate measures, mainly, the preparation of a new medicine and the communication of the occurrence of the error to the nurse or to the physician.

It is believed that the number of incidents that occurred in the neonatal unit may be greater than that reported, taking into account that there are errors that were not perceived by the professionals or were not recorded in the institution’s notification system. Thus, a combined approach of incident detection methods is considered to be the most complete and effective since these methodologies, when used alone, have some shortcomings. In order to achieve greater numbers of voluntary notifications, it is necessary to develop an effective safety culture, in which not only the institutional administration but also the care professionals are aware of their role in the development of harm reduction.
